# Understanding the role of wettability distribution on pore-filling and displacement patterns in a homogeneous structure via quasi 3D pore-scale modelling

**DOI:** 10.1038/s41598-021-97169-8

**Published:** 2021-09-08

**Authors:** Amir Jahanbakhsh, Omid Shahrokhi, M. Mercedes Maroto-Valer

**Affiliations:** Research Centre for Carbon Solutions (RCCS), School of Engineering and Physical Sciences, Heriot-Watt University, Edinburgh, UK

**Keywords:** Carbon capture and storage, Hydrogeology, Fluid dynamics, Hydrology

## Abstract

Most numerical simulation studies have focused on the effect of homogenous wettability on fluid flow dynamics; however, most rocks display spatially heterogeneous wettability. Therefore, we have used direct numerical simulations (DNS) to investigate wettability heterogeneity at pore-scale. We have built a quasi-3D pore-scale model and simulated two-phase flow in a homogenous porous media with homogenous and heterogeneous wettability distributions. Five different heterogeneous wettability patterns were used in this study. We observed that heterogenous wettability significantly affects the evolution of fluid interface, trapped saturation, and displacement patterns. Wettability heterogeneity results in fingering and specific trapping patterns which do not follow the flow behaviour characteristic of a porous medium with homogenous wettability. This flow behaviour indicates a different flow regime that cannot be estimated using homogenous wettability distributions represented by an average contact angle. Moreover, our simulation results show that certain spatial configurations of wettability heterogeneity at the microscale, e.g. being perpendicular to the flow direction, may assist the stability of the displacement and delay the breakthrough time. In contrast, other configurations such as being parallel to the flow direction promote flow instability for the same pore-scale geometry.

## Introduction

Immiscible fluids displacement in porous media is a complex phenomenon at both micro- and macro-scales, where different parameters from pore geometry, connectivity, and wettability, to viscosity ratio of invading and defending fluids, and relative magnitude of active forces (viscous, capillary and gravity forces) crucially influence the displacement mechanisms and fluid distributions^1–4^.

Experimental studies at pore-scale have demonstrated that wettability as a surface phenomenon has a significant impact on the evolution of invading (displacing) fluid, displacement patterns and distribution of fluids^5,6^. Therefore, the surface chemistry of the pore spaces (influenced by the spatial distribution of minerals and deposition of organic material deposited on the surface of minerals) is of great importance for characterizing the wettability of a system^7–20^.

Wettability is generally classified as homogeneous or heterogeneous^21^. For homogeneous wettability, the whole rock surface has a uniform molecular affinity for the fluids in contact. In contrast, for heterogeneous wettability, variation in affinities for the fluids at different regions is expected^21^. Most reservoirs display heterogeneous wettability, which can be categorized as either fractional or mixed-wet^22^. In the fractional-wet rock types, oil- and water-wet regions are randomly distributed with respect to the pore size, while in mixed-wet rock types, oil- and water-wet pores are mainly sorted based on their size (large pores are oil-wet and small pores are water-wet)^23^.

At the macro-scale, the bulk wettability of a porous medium is defined based on the wettability indices (e.g. the United States Bureau of Mines^24^ and Amott^25^) obtained from measured capillary pressure curves. The effect of wettability is usually incorporated in numerical reservoir simulations via flow functions, known as relative permeability (kr) and capillary pressure (Pc) curves obtained from laboratory measurements of core samples representing the same wettability conditions as the reservoir. Al-Khdheeawi et al. (2018) investigated the effects of macro-scale wettability heterogeneity and reservoir temperature on the efficiency of CO_2_ storage in saline aquifers^26^. They discretized a reservoir model to cells with dimensions of 43.2 × 36.4 × 8.8 m^3^ (in XYZ) and to mimic heterogeneous wettability, five sets of flow functions (strongly water-wet, weakly water-wet, intermediate-wet, weakly CO_2_-wet and strongly CO_2_-wet) were randomly assigned to different cells. Their simulation results showed that wettability heterogeneity accelerates the vertical CO_2_ migration and solubility trapping while reducing the residual trapping. These results endorse the significance of how to practically incorporate wettability heterogeneity in large scale subsurface geological models. To understand and precisely capture the effect of wettability heterogeneity on flow functions at the macroscopic level, it is essential to understand its role at the pore level. Zhao et al. adopted a lattice Boltzmann modelling approach to investigate the effect of wettability heterogeneity on relative permeability of oil/water two‐phase flow in porous media with different oil‐wet solid fractions ranging from 0.0 to 1.0. They observed that the relative permeabilities of both water and oil phases in porous media with heterogeneous wettability have quite different characteristics from those from porous media with homogeneous wettability^27^.

Recent developments in the in-situ contact angle measurements using X-ray micro-computed tomography (micro-CT) techniques have made the characterization of pore-scale wettability possible. Alhammadi et al. used X-ray micro-CT to obtain the distribution of contact angles at the pore-scale in calcite core samples^28^. They measured contact angle at hundreds of thousands of points for three rock samples after water flooding and found a wide range of contact angles both less than and greater than 90° (usually defined as mixed-wet conditions). AlRatrout et al. used the experimental results obtained by Alhammadi et al. to measure and investigate the impact of contact angle, interfacial curvature, and surface roughness on fluid flow in a mixed-wet rock sample^29^. They showed that rougher surfaces result in having lower contact angles and higher interfacial curvature for mixed wettability conditions.

Akai et al. investigated the impact of using non-uniform wettability distribution on relative permeability and fluid distribution during a water flooding experiment in a mixed-wet carbonate rock sample^28,30^. They used the colour-gradient lattice Boltzmann method to perform pore-scale numerical simulations. A non-uniform distribution of wettability obtained from the experiment performed by Alhammadi et al., and an automated algorithm developed by AlRatrout et al. were incorporated into their simulations. Additionally, they performed simulations using an average value of the measured contact angles as a uniform contact angle to compared with the non-uniform contact angle distributions and also with the experimental results^30,31^. In comparison with the experimental results, a much higher relative error was observed in water effective permeability for the case with a uniform contact angle distribution. They concluded that accounting for heterogeneous wettability distributions makes the pore-scale numerical simulation of waterflooding processes in mixed-wet rocks more reliable.

More recently Guo et al. investigated the impact of wettability heterogeneity on brine/supercritical CO_2_ relative permeability in sandstone rock samples^32^. Using X-ray micro-CT, they conducted in-situ measurements of contact angle in a Bentheimer sandstone core after CO_2_ flooding. A log-normal distribution of contact angle associated with a spatial correlation length was obtained. They observed that as the standard deviation and spatial correlation length of contact angle increase the variations in the relative permeability curves of both phases increase.

Micromodels have found a wide range of applications in studying various processes at pore scale (micron and even submicron)^33^. Morrow et al. performed micromodel experiments to study the effects of wettability change induced by crude oil on oil recovery and residual oil saturation^13^. Kim et al. observed changes in surface wettability of fused silica micromodel upon their reactions with supercritical CO_2_ and brine^34^. More recently, wettability effects on the displacement of brine by supercritical CO_2_ during drainage were investigated by Hu et al.^35^.

Different partially wetted materials and/or techniques to alter micromodel surface wettability have been used to mimic non-uniform wettability conditions at pore-scale. Schneider et al. and Schneider and Tabeling used UV-initiated graft polymerization of polyacrylic acid to fabricate polydimethylsiloxane (PDMS) micromodels reproducing a common wettability heterogeneity observed in many hydrocarbon reservoirs^3,36^. Lee et al. fabricated micromodels with cylindrical pillars with specific wettability (e.g., water-wet, oil-wet and intermediate) populated at different areas of the pore pattern^37^. They investigated the effect of wettability heterogeneity on fluid flow displacement, where water displaced decane or vice versa. It was observed that the impact of wettability is more significant as effective surface area (available solid surface to fluid/fluid interface) increases. Lee et al. fabricated glass micromodels with in-situ growing calcite pillars to replicate more realistic wettability and surface chemistry conditions^38^. Coating the surface of micromodels with specific minerals have been used to replicate surface wettability and chemistry of natural rocks. Their fabrication technique allows for direct observation of complex multiphase flows and geochemical interactions. Song and Kovscek investigated the effect of mixed wettability conditions on hydrocarbon recovery and improved oil recovery by low salinity waterflooding using a silicon micromodel with a coated surface by clay particles of kaolinite. Direct observation of the impact of fluid composition on the stability of kaolinite and the behaviour of clay was only possible using such a surface-functionalized micromodel^39^. Wang et al. coated glass micromodel channels with nanocrystals of calcite (CaCO_3_) and tuned the wettability of the calcite layer via an aging process to mimic carbonate rocks and study water/oil displacement^40^. Using spectroscopic techniques, e.g., fluorescence and Raman imaging, they observed and imaged in situ interaction of calcite and fluid in real-time. Most recently, Alzahid et al. presented a method to functionalize the pore pattern of PDMS micromodels with selective minerals such as quartz, kaolinite and calcite^41^. As a result, they fabricated a micromodel with roughness, contact angle, and wettability comparable to natural rocks. These micromodels allowed them to visually study the pore-scale flow mechanism of corner flow, leading to the snap-off of the non-wetting phase. Tanino et al. studied the effect of mixed-wet conditions on film flow and piston-like displacement in oil/water flow using a micromodel with a quasi-monolayer of crushed marble packed inside a channel^42,43^.

Although there has been significant progress in fabricating porous media replicas with more realistic spatial wettability configurations, it is not feasible to reproduce infinite possible distributions and perform laboratory experiments^33^. Pore-scale numerical simulations are very useful to study different scenarios and optimize the experimental design for further investigation. Most numerical simulation studies have investigated the effect of different uniform wettability on fluid flow dynamics, while most natural rocks display heterogeneous wettability (fractional or mixed-wet). Generally, the mixed-wet condition has been assumed for most pore-scale models^44,45^. However, the wettability distribution might be more complex^7,14,46,47^. Murison et al. studied the effect of wettability heterogeneity on immiscible displacement^47^. They observed that the spatial distribution of wettability has a strong influence on the fluid displacement process. Hiller et al. investigated the impact of wettability heterogeneity on capillary pressure curves and wettability indices^48^. They found that the wettability indices are not adequate to discriminate between different wettability heterogeneity cases. Both Murison and Hiller fabricated porous media replicas with fractional type of wettability heterogeneity where the distributions of wetting and non-wetting surface domains were not correlated to the local pore geometry. They also tried to isolate the effects of wettability heterogeneity from the effects of pore-scale geometry.

**Figure 1:**
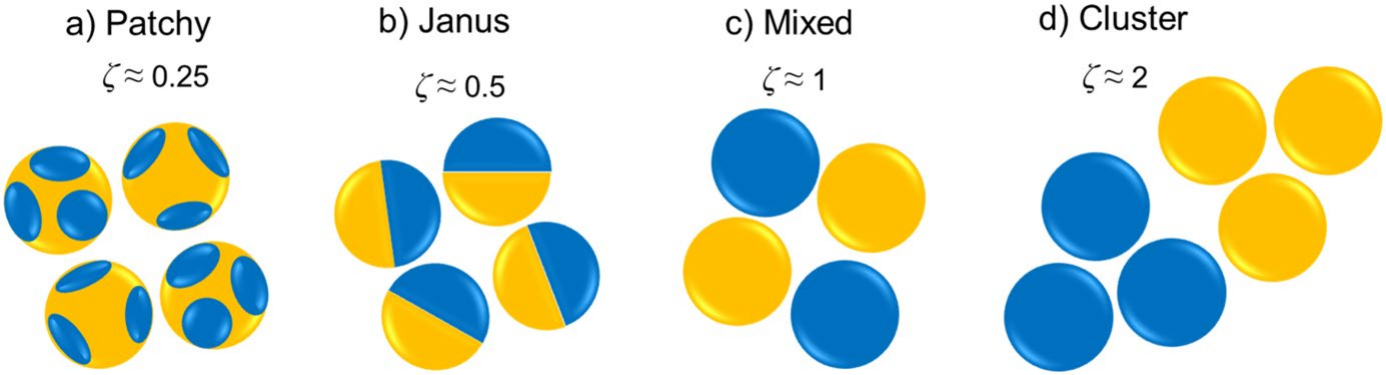
Sketch of four models for wettability heterogeneity. Wet- and non-wet surfaces are represented by blue and orange, respectively. All models have the same grain diameter and average surface energy but differ in the ζ (adapted from Murison/Hiller^7,47^).

Murison et al. and Hiller et al. used correlation length (*ζ*) to identify four different patterns of wettability heterogeneity^7,47^. The correlation length (*ζ*) is defined as the distance between a pair of surface areas with different wettability of i and j. This distance is usually normalized by the particle diameters (d). Figure 1 presents four different wettability patterns, namely Patchy, Janus, Mixed and Cluster.

Murison et al. and Hiller et al. created samples having equal surface areas with oil-wet and water-wet properties (50% oil-wet and 50% water-wet); however, the typical distance over which the wettability of the surfaces stayed unchanged within the sample was different^7,47^.

Numerical simulations help us overcome the limitations (technique, cost and time) associated with the fabrication of micromodels with different spatial wettability heterogeneity. In this work, we have used direct numerical simulations (DNS) to understand the complex effect of wettability heterogeneity on immiscible two-phase flow at the pore-scale. DNS studies were conducted using the Phase Field method using a commercial computational fluid dynamics (CFD) software (COMSOL Multiphysics®)^49^. We have built quasi-3D pore-scale models and simulated homogenous and heterogeneous types of wettability and investigated the effect of uniform and non-uniform wettability on the evolution of fluid interface, pressure behaviour of the system, displacement efficiency and trapped saturation in porous media. To ensure the effect of wettability distribution is isolated from other parameters, a uniform pore pattern is selected so the wettability of pores is not correlated with the size of the pores, and we consider no surface roughness. As wettability is classically defined using the concept of contact angle, we have employed the latter parameter to describe wettability in the models^7,44^. The novelty of this work is to systematically demonstrate the significance of incorporating distribution of contact angle (wettability) in numerical simulations in order to correctly predict the behaviour of multiphase flow processes, especially in the fractional and mixed-wet porous media. Moreover, the signature of both homogenous and heterogenous wettability distributions and their associated pore filling events on the global pressure drop across a homogenous porous structure was unveiled. We showed that the spatial distribution of wettability contributes to the front stability/instability in two-phase flow and can be as important as structural heterogeneity.

## Modelling theory and methodology

We performed Computational Fluid Dynamics (CFD) simulations using COMSOL Multiphysics® v.5.5, where the Cahn–Hilliard phase-field method was coupled with Navier–Stokes equation^49–51^. As a result, we obtained the velocity and pressure fields and the medium’s distribution of the phases. The phase field method (PFM) is a technique for capturing the interface between immiscible fluids. The interface transports with the flow while the total energy of the system is minimized. This approach ensures that there is a solution to flow in complex geometries without any simplification^52,53^. PFM has been used successfully for simulation of two-phase immiscible flow at pore-scale^54–56^. In this method, the interface between two fluids has a small but finite thickness (also known as diffuse interface), where the interfacial forces are smoothly distributed. The immiscible fluids are mixed and store a mixing energy inside the interface regions. A phase field variable (*φ*) was introduced by Yue et al. to define the concentration of fluids in which it varies continuously over the thin interface and remains uniform in the bulk phases^53^. In the case of two-phase flow, the two components’ relative concentrations in terms of *φ* are defined by (1 + *φ*)/2 and (1—*φ*)/2, respectively. The bulk of the phases have a fixed value, either *φ* = 1 or *φ* =  − 1, and the interface is presented by − 1 < *φ* < 1. Free energy of an isothermal two-phase system, when it is not at equilibrium conditions, can be written as a function of *φ*:

1 $$F\left[\varphi \right]={\int }_{\Omega }\left\{f\left(\varphi \left({\varvec{x}}\right)\right)+\frac{1}{2}\lambda {\left|\nabla \varphi \left({\varvec{x}}\right)\right|}^{2}\right\}d{\varvec{x}},$$

where *Ω* is the region of space occupied by the fluids in the system and ***x*** presenting the position in the region. The first term on the right-hand side is the bulk energy density *f(φ(x))* which has two minima corresponding to the two stable phases of the fluid, and the second term accounts for the surface energy. *f*(*φ(x)*) is defined as:

2 $$f\left(\varphi \left({\varvec{x}}\right)\right)=\frac{\lambda }{4{\varepsilon }^{2}}{\left({\varphi }^{2}-1\right)}^{2},$$

where λ is the magnitude of the mixing energy (a positive value), and ε is a capillary width representing the interface thickness. The surface tension (σ) relates these two parameters as shown in Eq. (3)^52,57^

3 $$\sigma =\frac{2\sqrt{2}}{3}\frac{\lambda }{\varepsilon },$$

By minimizing F[*φ*], the equilibrium interface profile of the system can be found^52^. This equation was generalized for transient (time-dependant) problems by Cahn and Hilliard. They approximated interfacial diffusion fluxes using the fact that they are proportional to chemical potential gradients^50–52^. The chemical potential *η* is defined as below, and by solving *η*(*φ*) = 0, the equilibrium interface profiles are obtained^52^.

4 $$\eta \left(\varphi \right)=\frac{\delta F\left[\varphi \right]}{\delta \varphi \left({\varvec{x}}\right)}={f}^{^{\prime}}\left(\varphi \left({\varvec{x}}\right)\right)-k{\nabla }^{2}\varphi \left({\varvec{x}}\right).$$

The convective Cahn–Hilliard equation can be written as:

5 $$\frac{\partial \varphi }{\partial t}+\overrightarrow{u}\cdot \nabla \varphi =\nabla \cdot \left[\gamma \nabla \left(\eta \right)\right],$$

where u is the velocity field, and γ is the mobility parameter which is defined as:

6 $$\gamma =\chi {\varepsilon }^{2},$$

In this equation, χ is the mobility tuning parameter that determines the Cahn–Hilliard diffusion’s time scale. χ must be high enough to keep the interface thickness constant and low enough not to damp the flow (convective motion).

The fluid flow is described by the incompressible Navier–Stokes equations with variable viscosity and density coupled with a phase field-dependent surface tension force^52,58^.

7 $$\nabla \cdot {\varvec{u}}=0,$$

8 $$\rho \left(\varphi \right)\left(\frac{\partial {\varvec{u}}}{\partial t}+{\varvec{u}}\cdot \nabla {\varvec{u}}\right)=-\nabla p+\nabla \cdot \mu \left(\varphi \right)\left(\nabla {\varvec{u}}+\nabla {{\varvec{u}}}^{T}\right)+\eta \left(\varphi \right)\nabla \varphi ,$$

where *p* is the pressure, *ρ*(*φ*) is the density, *μ*(*φ*) is the viscosity and *η*(*φ*)∇*φ* is the surface tension force. Both density and viscosity are defined to be constant in bulk phases and vary smoothly across the interface. Any physical properties of fluids are defined as:

9 $$\vartheta \left(\varphi \right)=\frac{\left(1+\varphi \right)}{2}{\vartheta }_{1}+\frac{\left(1-\varphi \right)}{2}{\vartheta }_{2},$$

where *ϑ*_1_ and *ϑ*_2_ are the physical properties of fluid 1 and 2, respectively.

For the solid surfaces, the wetted wall is defined with no-slip conditions. Therefore, the fluid velocity is zero at no-slip boundaries, and there is no motion for the fluid–fluid interface on the wetted wall. The wetting property of the wetted wall is defined by the contact angle which is related to the capillary width and the gradient of the phase field variable by Eqs. (10) and (11), respectively.

10 $${{\varvec{n}}}_{wall}\cdot {\varepsilon }^{2}\nabla \varphi ={\varepsilon }^{2}\cos\theta \left|\nabla \varphi \right|,$$

11 $${{\varvec{n}}}_{wall}\cdot \gamma \nabla \left(\eta \right)=0,$$

where ***n***_*wall*_ is the normal vector to the wall and *θ* is the contact angle. Finally, the curvature of the fluid–fluid interface (*κ*) is determined as

12 $$\kappa =2\left(1-{\varphi }^{2}\right)\frac{\eta \left(\varphi \right)}{\sigma }.$$

### Model description

**Figure 2:**
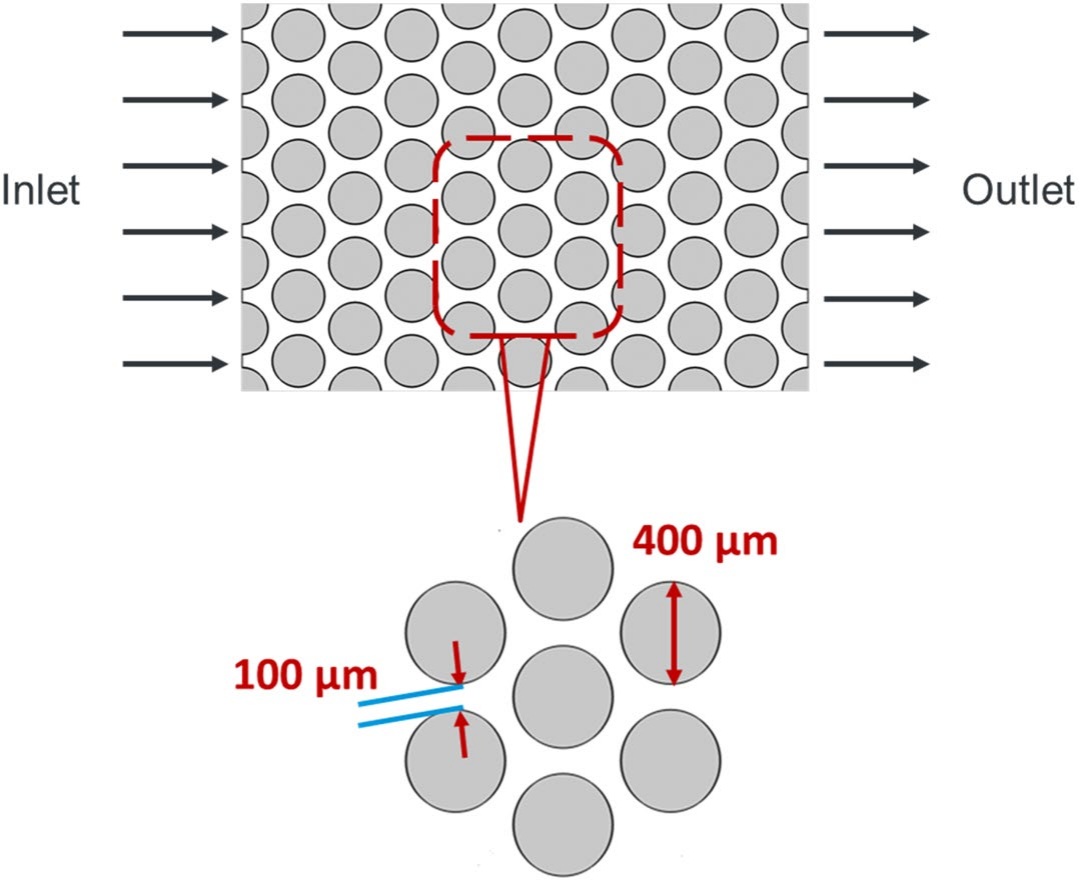
Representation of the geometry of the porous medium studied in this work.

The main objective of this work is to gain a fundamental understanding of the complex impact of wettability heterogeneity on immiscible two-phase flow. To isolate the effect of wettability heterogeneity on the displacement patterns and formation of fingers, we choose the simplest representation of a porous medium for numerical models that retains the most vital aspects of such a medium. A uniform 2D porous medium with the dimension of 4.33 (L) × 3 (W) mm, which consists of arrays of cylindrical solid pillars (representing grains), is used (Fig. 2). The pillars are placed on a regular triangular lattice to limit the number of the meniscus that can simultaneously invade into a pore to two menisci and retain the crucial aspect of cooperative pore filling^59,60^. The pillars have a diameter of 0.4 mm, and the width of the pore throats is 0.1 mm. The porosity (void fraction) of the medium is calculated at 42%.

Although considering a 3D pore geometry is more representative of natural porous media, this approach can hinder gaining basic insights on effective displacement mechanisms due to its complexity. However, assuming a constant height in the porous media, it is advantageous to model the system as a quasi-3D model. This quasi-3D model solves the 3D flow in the 2D plane by averaging the velocity variations in the height dimension. Moreover, using a quasi-3D model, the viscous damping of the fluid from the top and bottom of the channels is taken into consideration by adding a damping force term as follows^61^:

13 $${{\varvec{F}}}_{Da}=-\alpha {\varvec{u}}\boldsymbol{ }\,\mathrm{and }\,\alpha =\frac{12\mu }{{h}^{2}},$$

where *α* is the damping coefficient, *µ* is the volumetric average viscosity of present fluids, and *h* is the channel height. In this study, a constant depth of 0.02 mm is assumed for the porous media.

The simulation domain was discretized by triangular elements. The number of elements is nearly 700,000 (699,762) with the smallest size of 6 × 10^−5^ mm (0.06 μm) and the largest size of 5 × 10^−3^ mm (5.00 μm). Finer meshes were implemented around the boundaries and inside the throats and the pore bodies, as illustrated in Fig. S1 (see in Supplementary information). A mesh refinement study was performed to ensure that the simulation results do not change with further mesh refinement.

The porous pattern is initially filled with the defending fluid (water) with viscosity *μ*_*d*_ = 1.0 × 10^−3^ Pa s and density *ρ*_*d*_ = 1000 kg m^−3^, which is displaced by the invading fluid (air) with viscosity *μ*_*i*_ = 1.8 × 10^−5^ Pa s, density *ρ*_*i*_ = 1.2 kg m^−3^. The interfacial tension between the two fluids is *σ*_*air/water*_ = 72.86 × 10^−3^ N m^−1^.

The highest fluid velocity is attained at the narrowest channels in the pattern, and thus, the maximum values of Reynolds (Re) and Capillary (Ca) numbers are expected in these areas. The selection of injection velocity is dictated by the ranges of Re and Ca numbers of interest throughout this study. Based on the highest average velocity (u = 0.78 m s^−1^) in the throats, the Re number is in the range of 0.01 to 1.00 which guarantees to have laminar flow in the whole medium. The outlet of the model is kept at atmospheric pressure for all simulations.

Commonly, the capillary number (Ca), which is the ratio of the viscous to capillary forces, and the ratio of the defending to invading phase viscosities (M) are used for characterizing two-phase displacements where there is no gravitational force^56,62^. Lenormand et al. developed a displacement phase diagram to illustrate three basic displacement flow regimes of capillary fingering, viscous fingering and stable displacement for drainage processes based on Ca and M values^63^. Later, the phase diagram was refined by Zhang et al. (2011) with results of an experimental study on drainage processes in water-wet micromodels (Fig. S2, see in Supplementary information)^62^.

In an immiscible displacement process, when a less viscous fluid displaces a fluid of higher viscosity, the displacement front may be unstable resulting in viscous fingering^64^. Several parameters, such as injection rate, porous media heterogeneity and wettability, may contribute to the front instability. This study focuses on understanding the effect of wettability heterogeneity on front instability, trapped saturation, and displacement pattern at the pore-scale. We simulated displacements where air displaces water at log (Ca) =  − 3.7 ≈ − 4 and Log (M) =  − 1.73 ≈ − 2 conditions as shown in Fig. S2 (see Supplementary Information). Those displacements that represent a drainage process according to the surface wettability signify the viscous fingering regime based on the phase diagram of Zhang et al., while according to Lenormand et al., they are fitted in the transition zone between viscous fingering and stable displacement (Fig. S2).

We also examined the behaviour of uniform wettability distributions (constant contact angle) throughout the model. Three contact angles of 30°, 90° and 150° were used to represent strongly water-wet, neutral (intermediate)-wet and strongly non-water-wet conditions. These cases were used as references to evaluate profiling events, phase trapping and front propagation.

**Figure 3:**
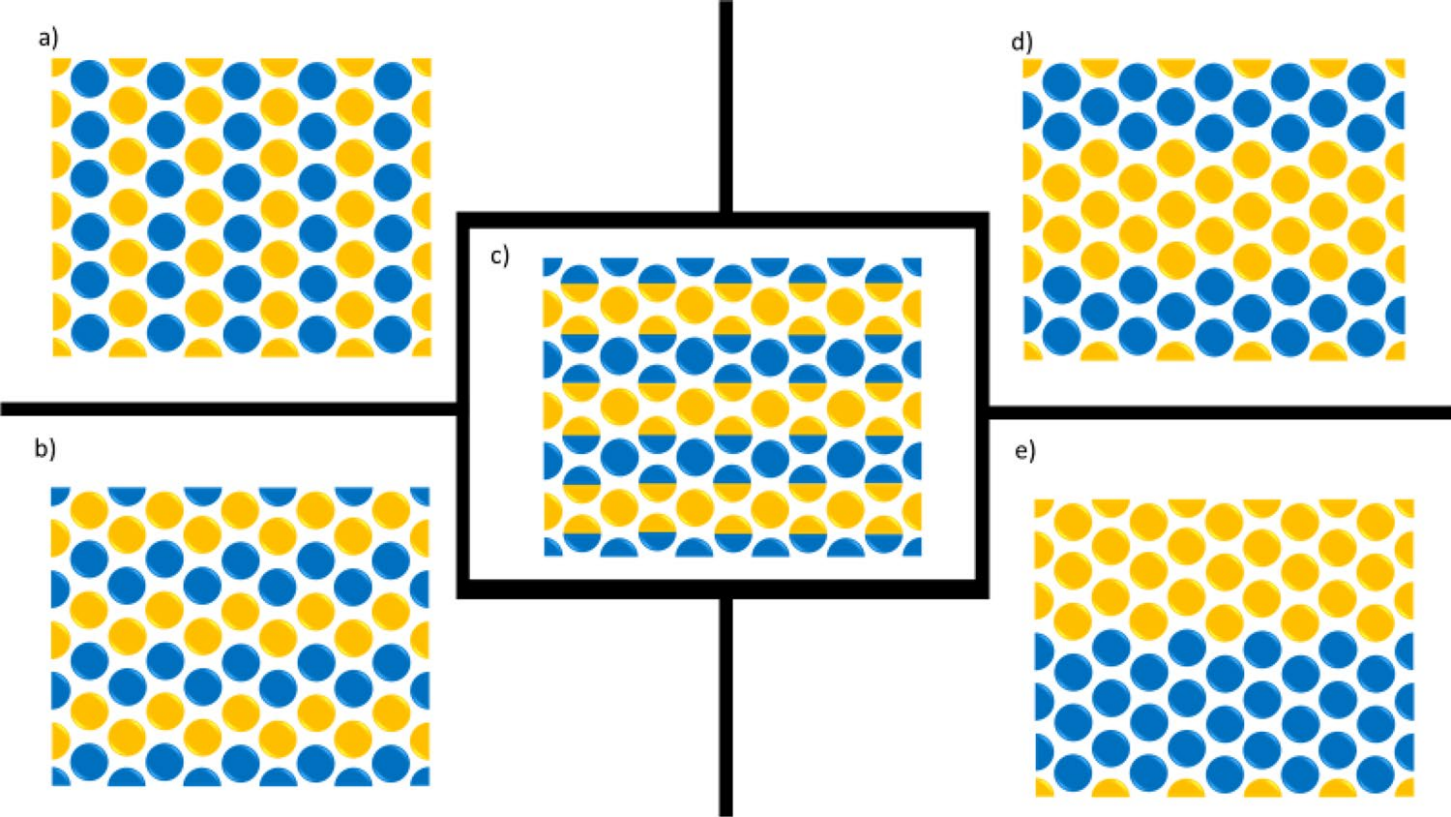
Five different wettability distribution patterns: (**a,b**) ζ = 1, (**c**) ζ = 0.5–1.5, (**d,e**) ζ = 1–2. Wet- and non-wet surfaces are represented by blue and orange, respectively.

In our numerical simulations with heterogeneous wettability conditions, we have taken a similar approach to Murison et al. and Hiller et al. and created porous media geometries exhibiting the same grain diameter and equal surface area for both different wettabilities (similar average surface energies) but differing in the correlation length *ζ* value^7,47^. Three Cluster type and two Mixed type patterns wettability distributions (as defined in Fig. 1) were used in the simulations (Fig. 3). Clusters samples were created by having clusters of hydrophilic and hydrophobic grains where the correlation length is larger than the grain diameter, e.g., *ζ* = 2. Mixed Samples that have smaller correlation length, equal to the grain diameter (*ζ* = 1), were created by having two neighbouring grains with different wettability, i.e. one hydrophobic and one hydrophilic^7,47^.

We used the static contact angle θ to define different wettability conditions in the simulation model. The effects of dynamic contact angle change and hysteresis are not taken into consideration in this study. Three different static contact angles of 30° (strongly water-wet), 90° (neutral) and 150° (strongly non-water-wet) are considered in this study.

## Results and discussion

In this section, we present the key results from the numerical simulations, focusing on the effect of homogeneous (uniform) and heterogeneous contact angle distribution on pore-filling events, trapped saturations and displacement patterns.

### Pore-filling events and trappings

During immiscible displacement, three main pore-scale front invasion events (a series of cooperative pore-filling events) have been observed and reported in the majority of experimental studies performed on micromodels and numerical modelling results at pore-scale^2,60,65^. Figure S3 (Supplementary Information) illustrates burst, touch and overlap events for a neutrally wet condition, where water displaces air through a stable displacement process.

**Figure 4:**
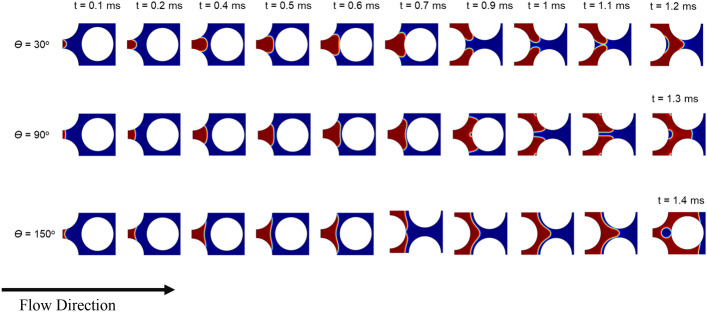
A sequence of pore-filling events (burst, touch and overlap) from left to right for a viscous fingering displacement of air (red) displacing water (dark blue). Top row: 30° contact angle through the denser phase represents strongly water-wet conditions or a drainage process, middle row: 90° contact angle represents intermediate wettability condition, and the bottom row: 150° contact angle through the denser phase represents strongly non-water-wet conditions or an imbibition process. Phase trapping was observed for all three contact angles, and in addition to the trapping due to menisci overlap, the touch event resulted in trapping at the upstream side of the pillar.

A sequence of pore-filling events is depicted in Fig. 4 for three different wettability conditions presented by contact angles of 30° (strongly water-wet), 90° (intermediate) and 150° (strongly non-water-wet) in a displacement process where air displaces water from left to right (log M =  − 2 and log Ca =  − 4). The fluid–fluid interface at the inlet throat develops a curvature corresponding to the given contact angle and moves from the throat toward the pore body. A convex meniscus is shaped for θ = 30° when the interface moves toward the pore body and touches the next pillar (t = 0.4–0.6 ms). The interface then splits into two menisci leading to fluid invading into the throats around the touched pillar (t = 0.7–1 ms). The two menisci will overlap (t = 1.1 ms) while moving to the next pore body and create one joined meniscus with a corresponding curvature to the contact angles. As the simulation results demonstrate, a water droplet is trapped at the downstream side of the pillar for the strongly water-wet conditions. Lee et al. performed a series of two-phase immiscible displacement experiments (water displacing decane and vice versa) in a microfluidic channel with a single cylindrical pillar. They investigated the effect of the wettability of the pillar on the displacement pattern and phase trapping. They observed that decane is entirely displaced by water in a water-wet micromodel; however, a droplet of decane adhered to the downstream side of the pillar in an oil-wet micromodel^37^. A similar respective observation was made in the experiments where decane displaces water. These observations validate the overlap trapping mechanism predicted by our numerical simulations at different wettability conditions for the drainage processes.

A similar wetting phase trapping mechanism due to the menisci overlap event (t = 1.3 ms) gets effective at θ = 90° and results in trapping a drop of defending phase to the downstream side of the pillar. A new trapping mechanism of intermediate and non-wetting phase arose due to the touch event at the upstream side of the pillar for θ = 90° and 150°, respectively. The capillary barrier, which is usually observed in the imbibition processes, is less effective at θ = 150° case, where air as the wetting phase with much less viscosity displaces water as the non-wetting phase^66–69^. The meniscus retained its concave curvature shape while passing through the junction of throat and pore and moving into the pore body. This curvature made the trapping possible when the interface touched the pillar (Fig. 4, θ = 150°, t = 1.1–1.4 ms). One other interesting observation during viscous fingering displacement (imbibition) at θ = 150° was that the overlap event happened ahead of the touch event, as shown in Fig. 4 at 0.7 ms.

Both trapping mechanisms of wetting and non-wetting phases, which were observed in Fig. 4 for drainage and imbibition processes, are schematically illustrated in Figs. S4 and S5 (Supplementary Information).

The pore-filling process in heterogeneously wetted structures, where individual pores have mixed wet surfaces, is a combination of different pore events (burst, touch and overlap). Figure S6 (Supplementary Information) illustrates the observed phase trappings as the result of pore filling events for five different wettability heterogeneity patterns. Although similar pore-filling events and phase trapping behaviours were observed for the homogenous wettability cases investigated (30°, 90° and 150° contact angles), the trapping was significantly irregular for all heterogenous wettability patterns. Larger continuous trapped ganglions were due to the spatial distributions of wettability.

### Displacement patterns

Our numerical simulation results discussed above have demonstrated that the sequence of pore invasion directly depends on the combination of viscosity ratio of the defending and invading phases, competition between viscous and capillary forces and the surface wetting properties. The pore-filling events consequently govern the type of displacement (piston-like or distorted front) and the phase trapping in the porous media. Despite the large volume of experimental data available from both micromodel and core flooding experiments, there are still conflicting evidence on the true effect of wettability on fluid displacement efficiency^70^. Recent developments in integrating microcomputed tomography (micro-CT) with core flooding experiments have revealed flow behaviour and fluid distribution at microscale^33^. However, in most cases, the sampled porous media have had heterogeneity level which may overshadow the effect of wettability and wettability heterogeneity. On the other hand, although micromodels have made this possible to have more control over the structural heterogeneity of porous media, further development in fabrication techniques is required to replicate wettability heterogeneity in micromodels. Therefore, to overcome these limitations, we use numerical simulation to look at the effect of wettability and wettability heterogeneity on fluid displacement processes in a deliberately selected homogenous and uniform porous structure.

#### Homogenous (uniform) wettability

**Figure 5:**
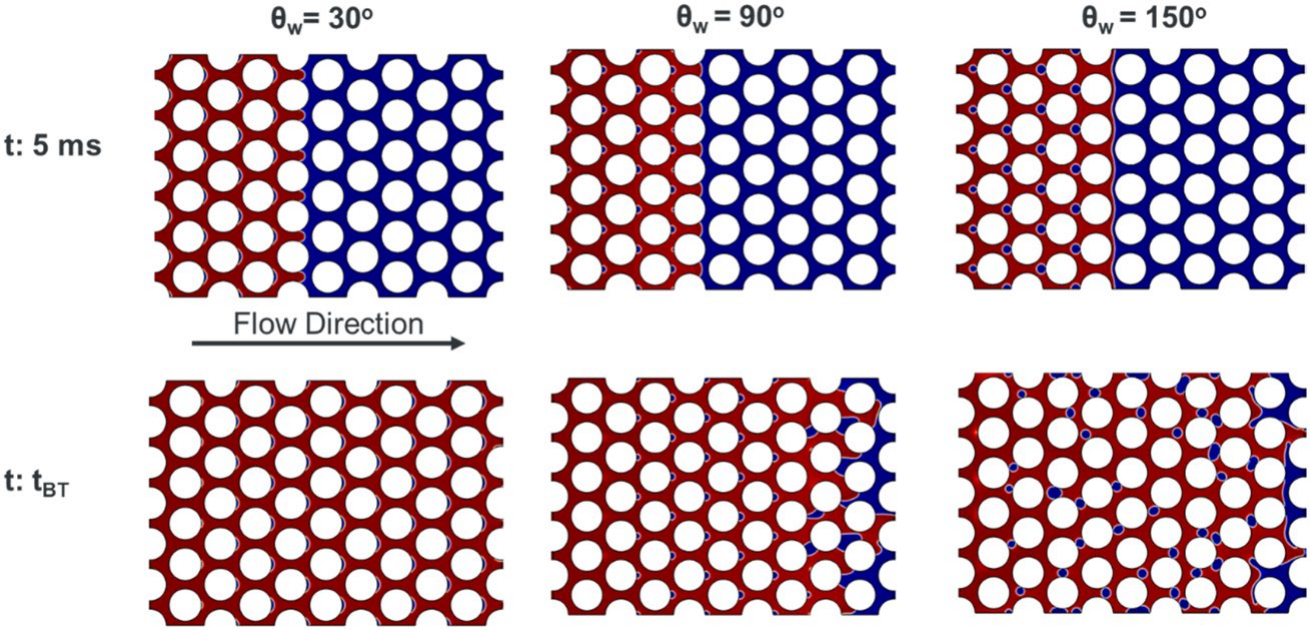
The dynamics of air (red) displacing water (dark blue) at log Ca =  − 4 and Log M =  − 2. The medium has homogeneous wettability presented by constant contact angle (30°, 90° and 150°) with respect to the defending phase.

The fluid displacement happened at viscous fingering regime, where air displaces water from left to right. The simulation results (Fig. 5) showed a stable front propagation for all three homogeneous wettability cases (constant contact angles). For θ = 30°, where air as the non-wetting phase displaces water as the wetting phase, the front is stable and the displacement is piston-like; however, water gets trapped at the downstream side of pillars as a result of overlap events. Zhang et al. observed a similar stable type of displacement associated with wetting-phase trapping when they displaced a wetting phase by a non-wetting phase at Log Ca and Log M of − 2.87 and − 1.95, respectively^62^. Although the displacement conditions in our study are in the range of viscous fingering regime according to the displacement phase diagram since the porous media is homogenous, no fingering was observed. However, phase trapping occurred in all three cases as a result of both overlap and touch events. The trapped phase at 90° and 150° water contact angles is either neutral or non-wetting phases, respectively, and therefore, there is a higher chance that the trapped bubbles detached from the pillar surface and mobilize. The detachment of the bubbles can be the main reason behind having a more distorted interface and trapping pattern for these two cases at the breakthrough time, as shown in Fig. 5.

**Figure 6:**
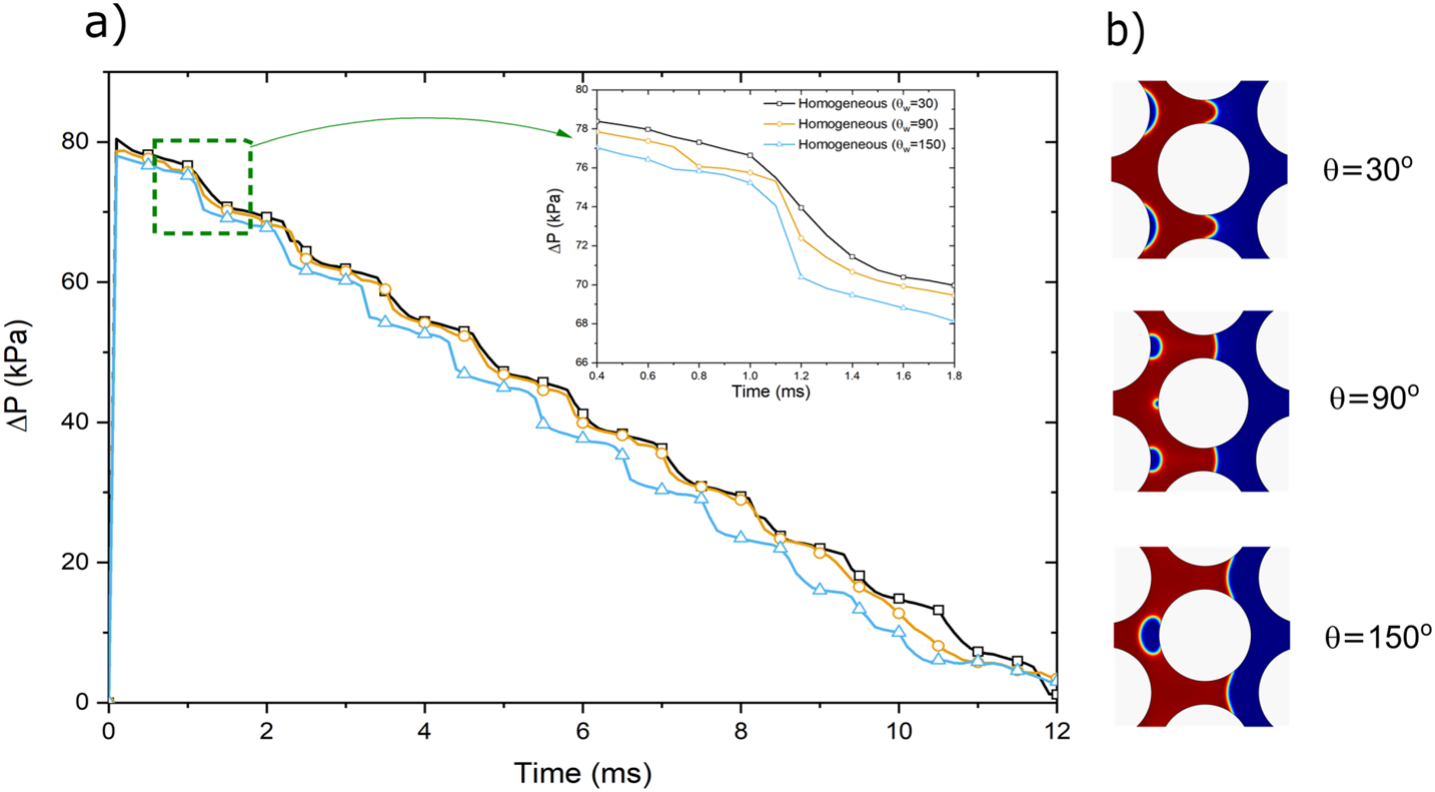
(**a**) Pressure drop (kPa) across the porous medium for homogeneous wettability at three different contact angles (30°, 90° and 150°) during displacement at log Ca =  − 4 and log M =  − 2. (**b**) The corresponding pore filling and trapping events of stepwise pressure drops (one is shown the dotted green box).

As expected, the displacement at θ_w_ = 30°, which is a drainage process, where the non-wetting phase (air) displaces the wetting phase (water), shows the highest pressure drop across the medium among all three cases (Fig. 6). The lowest pressure drop is for the imbibition process (displacement at θ_w_ = 150°), and the displacement at θ_w_ = 90° fits in between drainage and imbibition. The signature of quick pore-filling events (e.g. Haines jumps) and the corresponding phase trapping is clearly reflected on the pressure drop curves as a stepwise reduction, shown in Fig. 6. The dashed green box magnifies the step reduction in pressure drop curves for all three contact angles (Fig. 6a) and their corresponding pore events, and trapping mechanisms are illustrated in Fig. 6b for all three contact angles.

#### Heterogeneous wettability

The structural and chemical complexity of real geomaterials is often oversimplified to study fluid flow in porous media at the core scale. It is essential to perform experiments and numerical simulations that better represent the complexity of natural porous media and validate the simplifying assumptions. In this section, we investigate the effect of wettability heterogeneity on fluid displacement processes, while the structural heterogeneity is kept out of the equation by using a homogenous porous structure in our numerical simulations.

**Figure 7:**
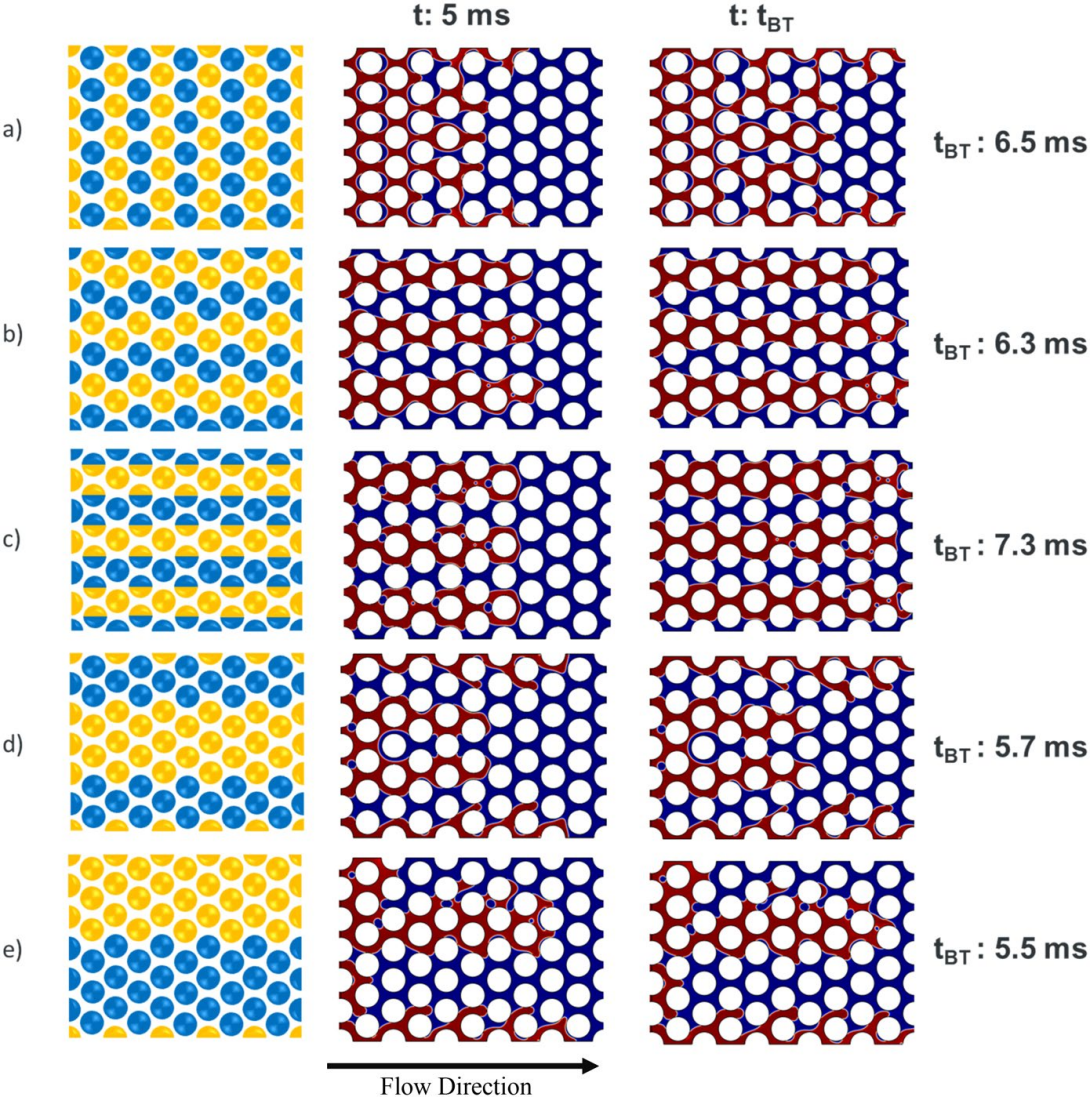
The dynamics of air (red) displacing water (dark blue) at log Ca =  − 4 and log M =  − 2 for five porous media with different wettability heterogeneity patterns.

Figure 7 depicts the fluid displacement and saturation distribution at two different times of 5 ms and breakthrough time (t_BT_) for each heterogeneous wettability pattern (videos of the dynamic displacement simulations shown in Fig. 7 are available in Supplementary Information). Similar to the numerical simulation of homogeneous wettability cases, the displacement happened at viscous fingering regime (log Ca =  − 4 and log M =  − 2). Apart from the pattern “a” (mixed type), the rest of the heterogeneous wettability patterns have distribution directions parallel to the flow. In contrast, the heterogeneous pattern of wettability in pattern “a” led to lateral spreading of the front and hindered finger generation compared to other patterns. The lateral wettability distribution resulted in significantly different saturation distribution and trapping between pattern “a” and the rest of patterns. A prevalent observation among pattern “b” to “e” is the significant importance of wettability heterogeneity pattern on developing viscous fingering during the displacement. Analogous to porous media heterogeneity, where the difference in permeability creates preferential paths through high permeable areas, the heterogeneity in wettability provides paths for the invading fluid to move throughout the porous structure. As the simulation results demonstrate, the development of fingers leaves a large area of porous media unswept, creates disconnected ganglia of defending phase and results in early breakthrough time and less displacement efficiency (lower recovery).

As previously stated, both contact angles (30° and 150°) have the same surface area in all heterogeneous wettability patterns. Although similar pore-filling events were observed, the overall fluid flow behaviour of heterogenous models are substantially different from their counterpart homogenous model with the average contact angle of 90°. Moreover, despite the homogenous cases, where mainly piston-like displacement was observed, front instability is the main characteristic of heterogeneous cases. These results confirm that homogenous wettability represented by an average contact angle cannot simply represent heterogeneous wettability conditions in natural porous media.

**Figure 8:**
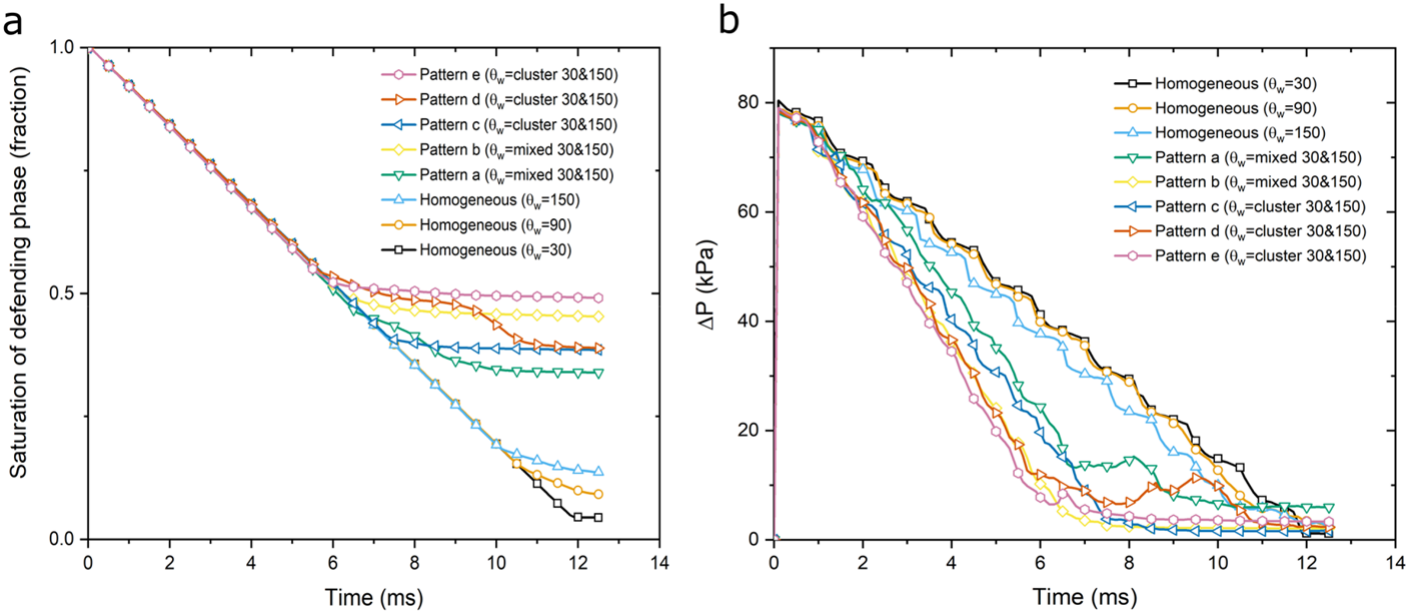
(**a**) Saturation of the defending phase and (**b**) Pressure drop (kPa) across the porous medium for all simulation cases (homogeneous and heterogenous wettability patterns) during displacement at log Ca =  − 4 and log M =  − 2.

Figure 8 compares the variations in the saturation of defending fluid and pressure drop for both homogenous and heterogeneous wettability cases. Considering that homogenous models showed a piston-like type of displacement, higher efficiency was expected and observed compared to the heterogeneous models. However, among the homogenous cases, the drainage process where air displaced water at a water contact angle of 30°, the highest displacement efficiency was predicted. The residual saturations of all heterogeneous models were higher than 30% by the time of 12 ms which was significantly higher than that of all three homogenous wettability models.

Interestingly, lower pressure drops across the porous medium were observed for all different heterogeneous wettability patterns compared to the homogenous wettability models. The preferential paths introduced by the wettability heterogeneity imposed less viscous pressure drop requirement to displace the resident fluid in the porous medium. Evidently, the intermediate homogenous wettability model with 90° contact angle showed significantly different recovery and pressure drop compared to the heterogeneous wettability models.

## Conclusions

We have investigated the influence of surface wetting properties and wettability heterogeneity on the two-phase flow of air and water at the pore-scale using direct numerical simulations. A series of numerical simulations with different homogeneously or heterogeneously distributed contact angles have been carried out to understand two-phase flow at a specific viscosity ratio and capillary number in a homogenous porous medium. We observed pore-scale phase trapping mechanisms corresponding to pore-filling events. We concluded that heterogenous wettability plays a substantial role in front evolution, phase trapping and displacement efficiency. Wettability heterogeneity affects the breakthrough time and, in particular, the saturation distributions. Different wettability distributions result in different trapping patterns as well as front instability. The residual saturation of all heterogeneous models was higher than that of all three homogenous wettability models. Some wettability distributions parallel to the flow direction, and *ζ* > 1 trigger front instability and the creation of viscous fingers. On the other hand, wettability distributions perpendicular to the flow direction may promote the evolution of a more stable front. Our numerical simulations showed that a porous medium with equal surface areas of two different contact angles (or wetting properties) does not simply behave akin to a medium with its average contact angle. In other words, intermediate or neutrally wet porous media usually represented by a contact angle of 90° do not behave similar to porous media with heterogeneously distributed surfaces with similar average wetting conditions, e.g., 30° and 150°.

Finally, although structural heterogeneity of the porous media mainly affects the fingering phenomena, our study shows that, in most cases, the wettability heterogeneity can result in viscous fingering even in a homogeneous system.

## Supplementary Information

Supplementary Information.

Supplementary Video 1.

Supplementary Video 2.

Supplementary Video 3.

Supplementary Video 4.

Supplementary Video 5.
