# Supplementary material for: Understanding the role of wettability distribution on pore-filling and displacement patterns in a homogeneous structure via quasi 3D pore-scale modelling
Source: Sci Rep. 2021 Sep 8;11:17847. doi: 10.1038/s41598-021-97169-8 (PMC8426499; doi:10.1038/s41598-021-97169-8)
Supplement: Supplementary file 1 — Supplementary Information. [file 41598_2021_97169_MOESM1_ESM.docx]

**Understanding the role of wettability distribution on pore-filling and displacement patterns in a homogeneous structure via quasi 3D pore-scale modelling**

**Amir Jahanbakhsh*, Omid Shahrokhi, M. Mercedes Maroto-Valer**

Research Centre for Carbon Solutions (RCCS), School of Engineering and Physical Sciences

Heriot-Watt University, Edinburgh, UK

*a.jahanbakhsh@hw.ac.uk


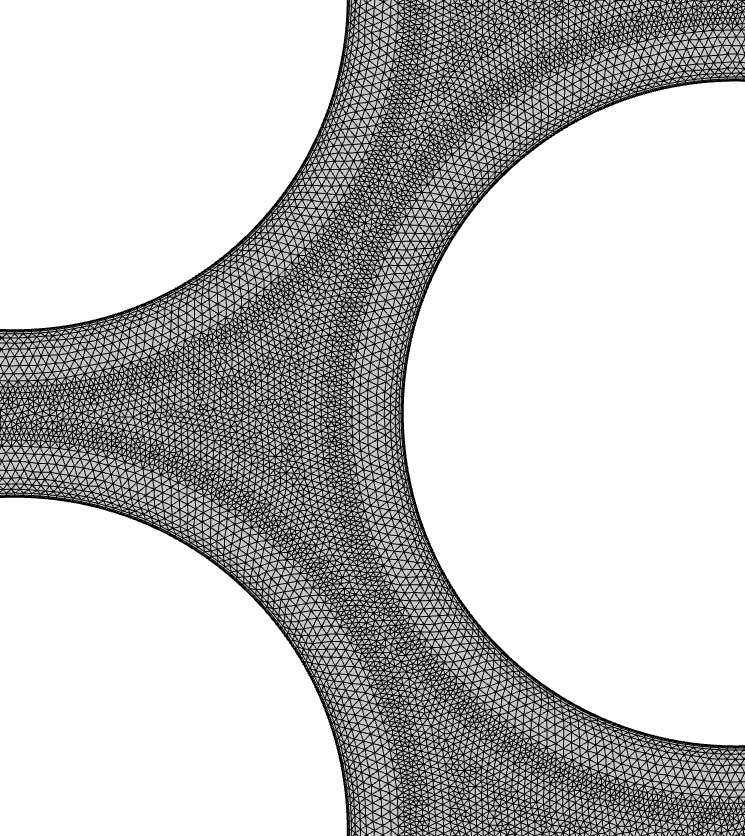


Figure S1: Mesh elements in an enlarged section of the simulated domain containing parts of pillars, pores and throats. The maximum element size is 0.005 mm (5 micrometres), and the mesh is refined near the boundaries to capture the contact line movement adequately.

**
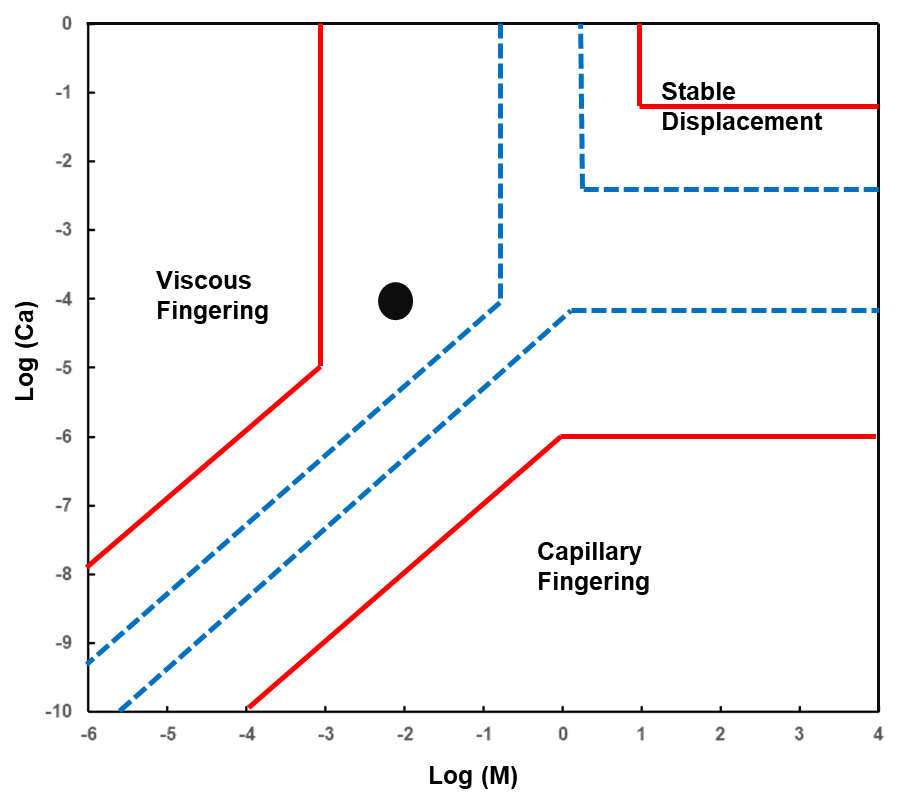
**

Figure S2: Displacement phase diagram in the log (M) –log (Ca), the red lines depict the regime boundaries according to Lenormand et al. (1988), whereas the blue dash lines are the extended regime boundaries based on Zhang et al. (2011). The black circle indicates the conditions simulated in this study.


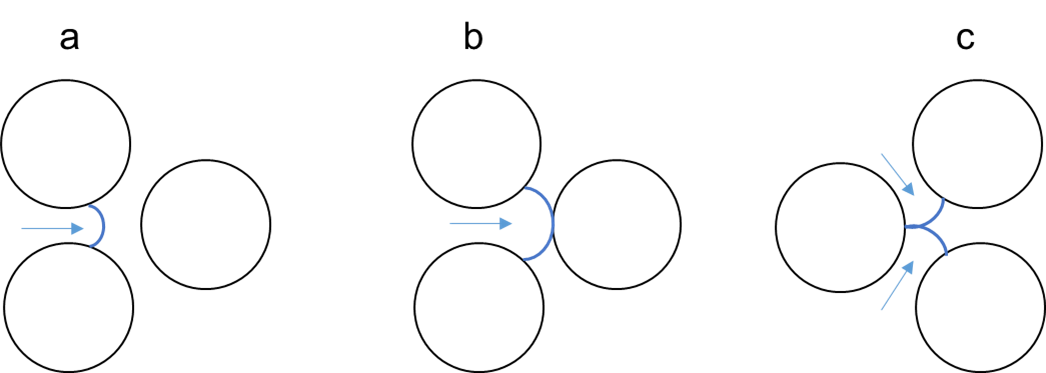


Figure S3: Cooperative pore-filling events: (a) Burst: a meniscus (unstable interface) unpins from a pore throat and advances into the pore. (b) Touch: a meniscus touches the nearest grain, often splitting into two menisci right after the touch. (c) Overlap: two menisci merge and form a new meniscus


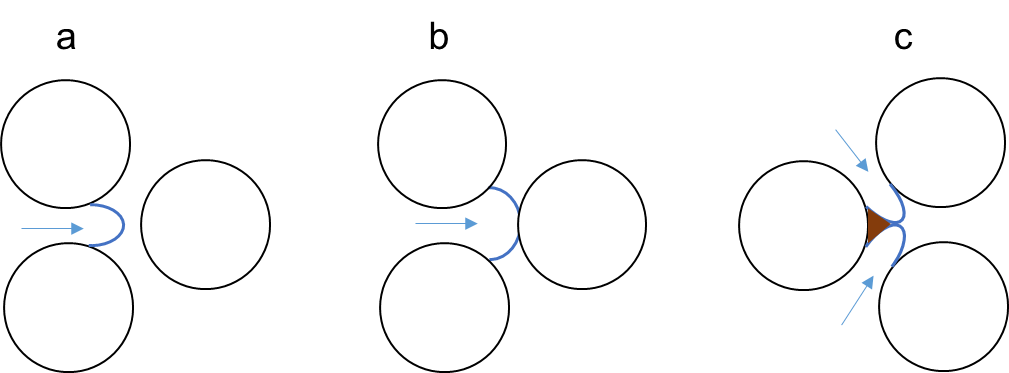


Figure S4: Schematic of wetting (and intermediate) phase trapping because of overlap events during a drainage process. (a) Burst event (b) Touch event (c) Overlap event and associated phase trapping.


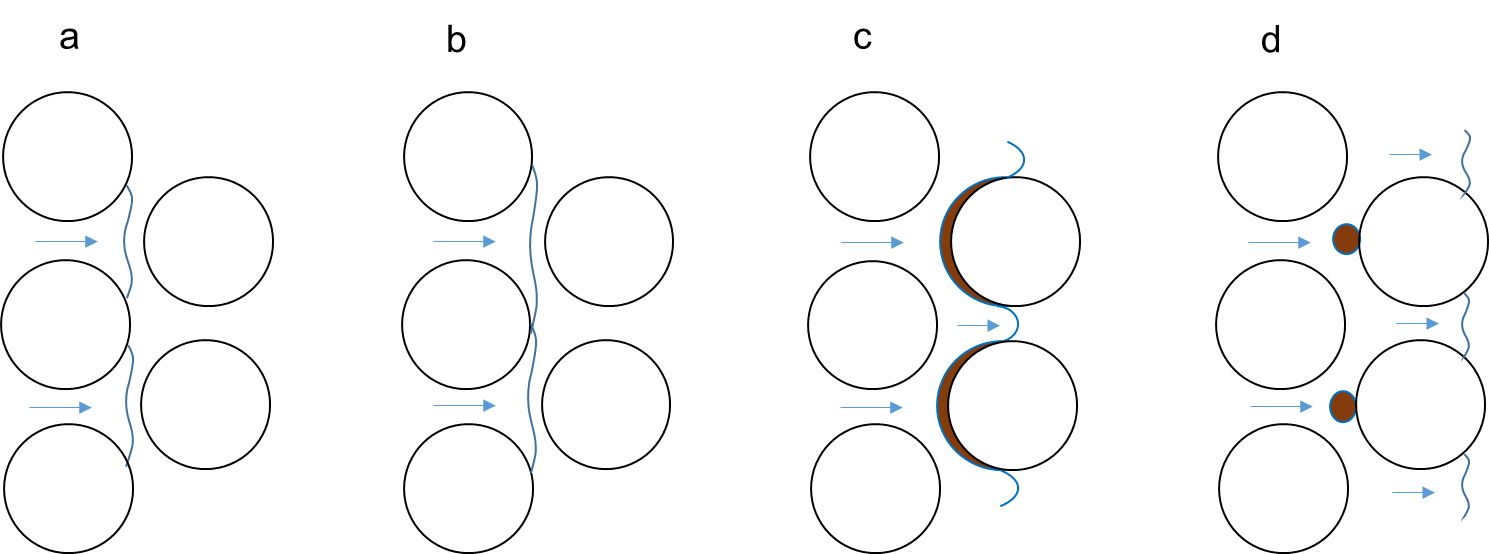


Figure S5: Schematic of non-wetting (and intermediate) phase trapping because of the touch event during an imbibition process. (a) Burst event (b) Overlap event (c) Touch event (d) Phase trapping due to touch event.


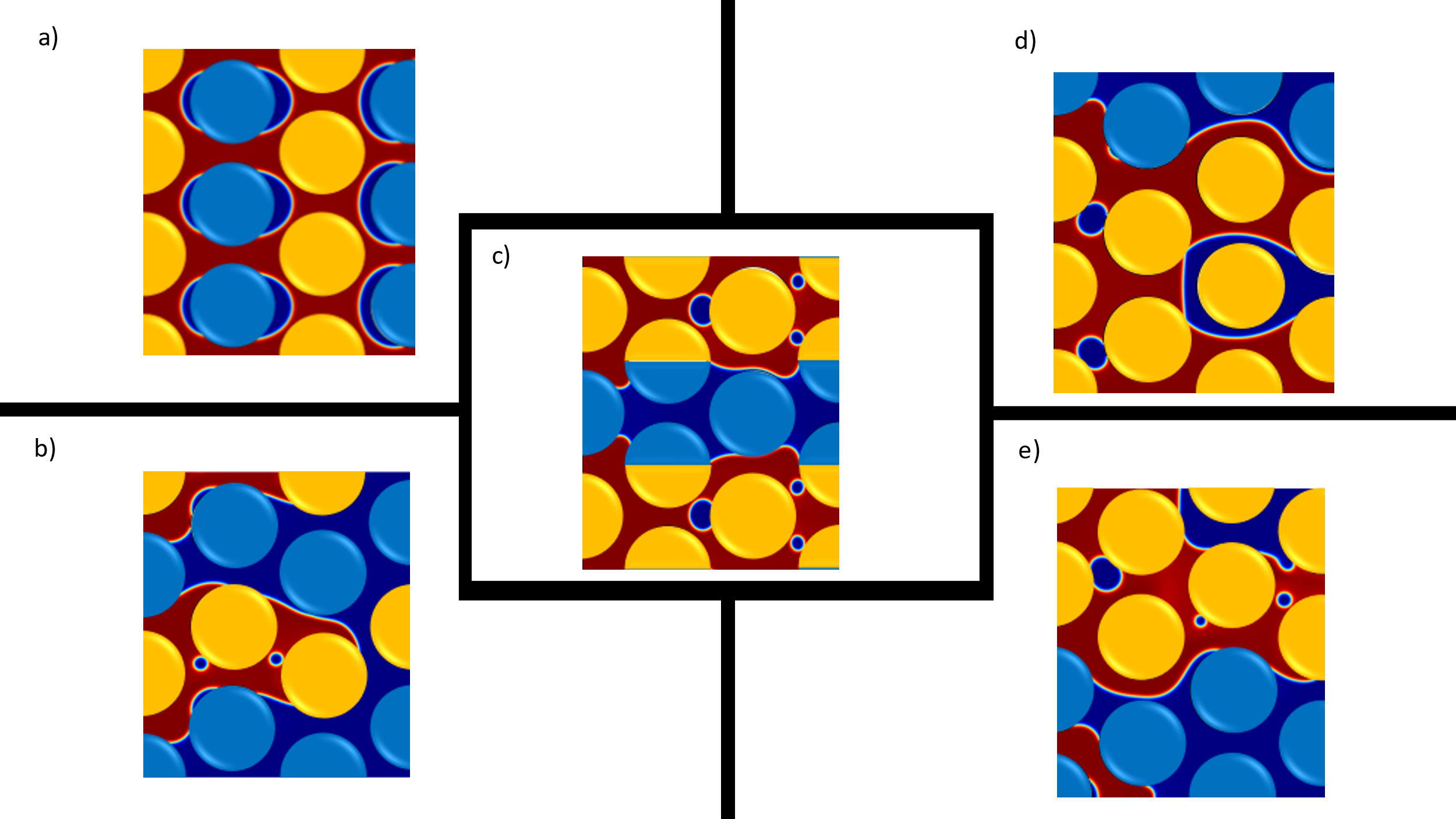


Figure S6: Phase trappings observed as the result of pore filling events for five porous media with different wettability heterogeneity patterns a, b) ζ =1 c) ζ = 0.5-1.5, d and e) ζ =1-2. Water- wet (30^o^) and non-water-wet (150^o^) surfaces are represented by blue and orange, respectively. Air (red) displacing water (dark blue).

Video files: The provided video clips show the dynamics of air (red) displacing water (dark blue) at log Ca=-4 and log M= -2 for five porous media with different wettability heterogeneity patterns (from “a” to “e” shown below).

**
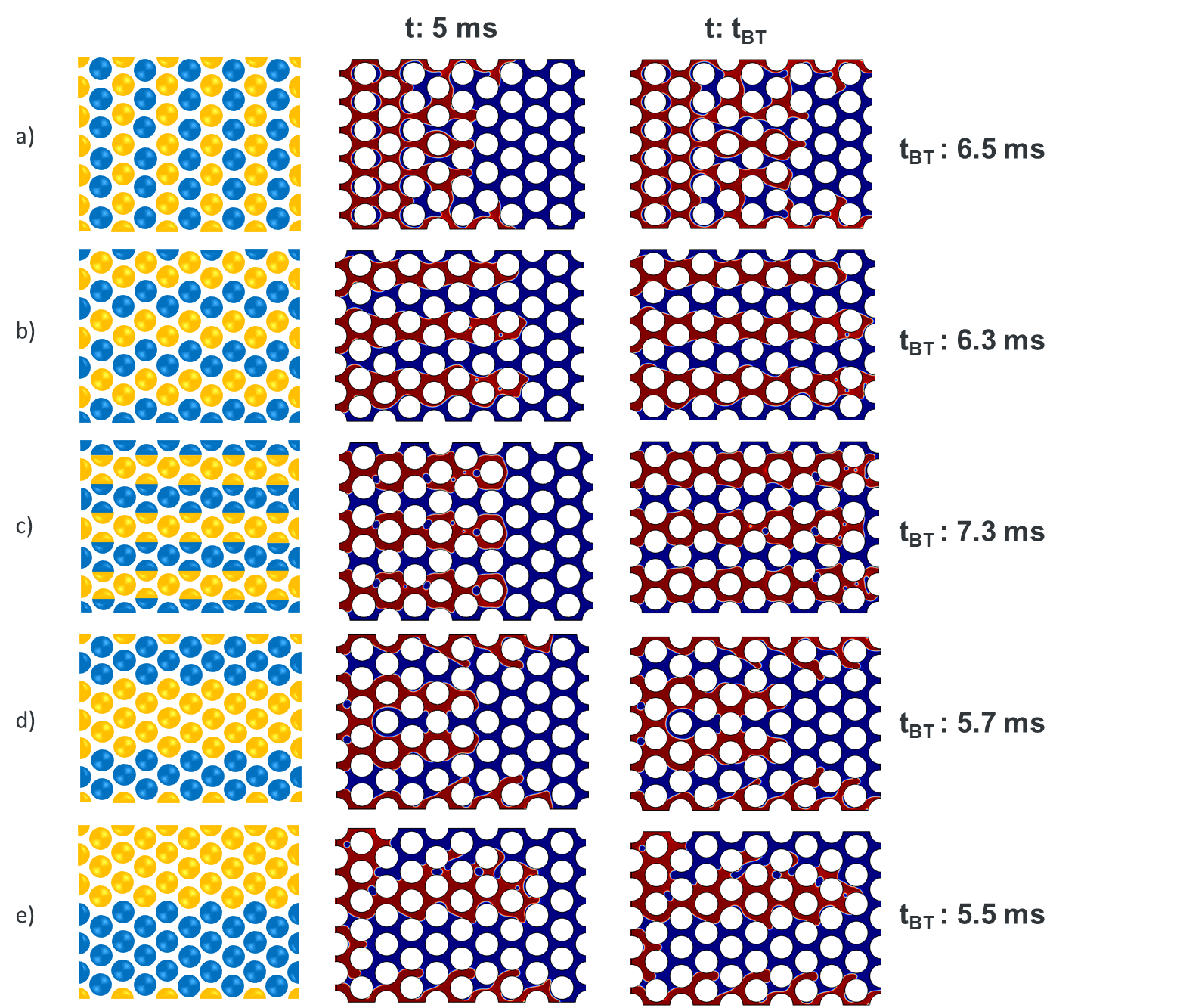
**
